# Marine-Inspired Drugs and Biomaterials in the Perspective of Pancreatic Cancer Therapies

**DOI:** 10.3390/md20110689

**Published:** 2022-11-01

**Authors:** Andreia S. Fernandes, Catarina Oliveira, Rui L. Reis, Albino Martins, Tiago H. Silva

**Affiliations:** 1 3B’s Research Group, I3Bs–Research Institute on Biomaterials, Biodegradables and Biomimetics, University of Minho, Headquarters of the European Institute of Excellence on Tissue Engineering and Regenerative Medicine, AvePark, Parque de Ciência e Tecnologia, Zona Industrial da Gandra, Barco, 4805-017 Guimarães, Portugal; 2 ICVS/3B’s–PT Government Associate Laboratory, Braga, 4710-057 Guimarães, Portugal

**Keywords:** marine natural products, marine biomaterials, drug delivery, pancreatic cancer, anti-tumour, biodiscovery

## Abstract

Despite its low prevalence, pancreatic cancer (PC) is one of the deadliest, typically characterised as silent in early stages and with a dramatically poor prognosis when in its advanced stages, commonly associated with a high degree of metastasis. Many efforts have been made in pursuing innovative therapeutical approaches, from the search for new cytotoxic drugs and other bioactive compounds, to the development of more targeted approaches, including improved drug delivery devices. Marine biotechnology has been contributing to this quest by providing new chemical leads and materials originating from different organisms. In this review, marine biodiscovery for PC is addressed, particularly regarding marine invertebrates (namely sponges, molluscs, and bryozoans), seaweeds, fungi, and bacteria. In addition, the development of biomaterials based on marine-originating compounds, particularly chitosan, fucoidan, and alginate, for the production of advanced cancer therapies, is also discussed. The key role that drug delivery can play in new cancer treatments is highlighted, as therapeutical outcomes need to be improved to give further hope to patients.

## 1. Introduction

Our planet is covered predominately by water and marine biodiversity is unrivalled. Currently, modern technologies make it possible to attain unexplored sea depths, making marine biota more and more available to researchers. Over the years, marine resources have gained increasing attention from biomedical research. Up to now, around 28,000 new compounds of marine origin have been discovered [1]. These natural marine compounds could play a key role in cancer research, as they are usually less toxic than conventional chemotherapy agents, effective, inexpensive, and, in most cases, easily available [2]. They may be used to inhibit cancer development, progression, and metastasis [3]. Several marine-derived metabolites can inhibit tumour cells’ growth both in vitro and in vivo, as well as in cancer clinical trials [4]. The exploitation of marine resources could open the doors to a new generation of anticancer drugs, with a positive impact on millions of lives. 

Even with all the scientific advances, cancer remains one of the main causes of death worldwide. Cancer is a highly heterogeneous disease at the molecular, cellular, tissue, and organ levels, which allows for progression and the evasion of available therapies. Chemoresistance, defined as the ability of cancer cells to evade and survive in the presence of therapeutics, is the main problem in cancer patients’ treatment [5,6]. For this reason, the main goals of current oncology treatments are to find efficient compounds/systems capable of attenuating the critical side effects caused by conventional therapies and, in particular, new strategies that can overcome the problem of resistance to antineoplastic drugs. 

Compared with other cancers, pancreatic cancer (PC) has a low incidence in the population, but it remains one of the deadliest cancers, with a poor prognosis, particularly in advanced stages. For this reason, it ranks as the fourth or fifth most common cause of cancer death in developed countries [7,8]. Chemoresistance has become the main problem in this cancer management, supporting the immediate need for more efficient therapies. Novel molecules inspired by marine natural products are now available for the development of new approaches to treat this particular cancer. In this review, a general overview of the most promising molecules and drugs for PC, inspired by marine compounds, will be discussed. Moreover, different biomaterials, advanced therapies, and models based on marine-derived compounds promising to enhance the understanding of the disease and the outcome of the therapeutic approach will be addressed. The main goal of this review is to highlight the great value of marine ecosystems for human life, considering always the importance of their preservation and conservation, as a healthy ocean contributes to a healthy society and all together to a healthy world.

## 2. Pancreatic Cancer: A Silent Killer

Pancreatic cancer epidemiology is well documented in the literature, but its aetiology is complex and multifactorial. Several genetic and environmental factors are associated with this cancer. Diabetes, chronic pancreatitis, and somatic mutations are some possible identified risk factors for this disease [9,10]. Indeed, PC is uncommon, but it has an exceptionally high mortality rate. Diagnosis is highly challenging because, in the first stages, patients do not present or show any specific symptoms. Consequently, many cases are only identified at a later stage, with an extremely poor prognosis since the cancer is advanced and has usually metastasised, with only 10–15% of them being surgically resected. Pancreatic ductal adenocarcinoma (PDAC) is measured as the most common pathological type of PC, representing about 94% of the associated cases with the worst survival [11,12].

### 2.1. Current Treatments and Challenges

The treatment for PDAC is dependent on several criteria, including tumour size, lymph node involvement, stage of infiltration into adjacent tissues, and presence of metastasis [13]. When the cancer is localised, surgical resection is the first-line treatment, remaining the only suggestion for a cure. Unfortunately, PDAC is diagnosed in advanced stages in 95% of patients, and in most of them, surgery brings contradictions. Since it can only be performed in 15% of patients and many of them experience recurrences after surgery, this treatment option represents a low cure rate. However, resective surgery does not exclude the use of adjuvant chemotherapeutic treatment [14,15,16].

For patients with advanced disease with metastatic PDAC, both chemotherapy and radiotherapy are considered the standard treatment approaches. Several drugs are available and approved as first-line treatment options, such as gemcitabine (GEM) and erlotinib [17]. GEM, an analogue of the pyrimidine nucleotide deoxycytidine, is usually administered in higher and repeated doses and is associated with many negative side effects [18]. Different studies reported cancer cells’ resistance through many different but unclear mechanisms of this drug [18,19,20]. As second-line therapies, several chemotherapeutic agents have shown efficacy in the treatment of PDAC, such as the combination of GEM with any of the following drugs: fluorouracil (5-FU), capecitabine, pemetrexed, topoisomerase inhibitor, irinotecan and exactecan, platinum compounds (cisplatin and oxaliplatin), and taxanes (paclitaxel and docetaxel) [21,22,23].

The treatment of PDAC is extremely difficult, with none of these approaches presenting a significantly increased survival rate. The poor efficacy of these treatments, undetectable metastases, and the development of chemoresistance are the main causes of these dismal results. Other treatment approaches have emerged and attracted the attention of the medical community such as target, gene, and immunotherapies but more studies are needed before they can become available as standard treatment options.

### 2.2. The Pancreatic Cancer Ecosystem as a Potential Therapeutic Target against Chemoresistance

**Figure 1:**
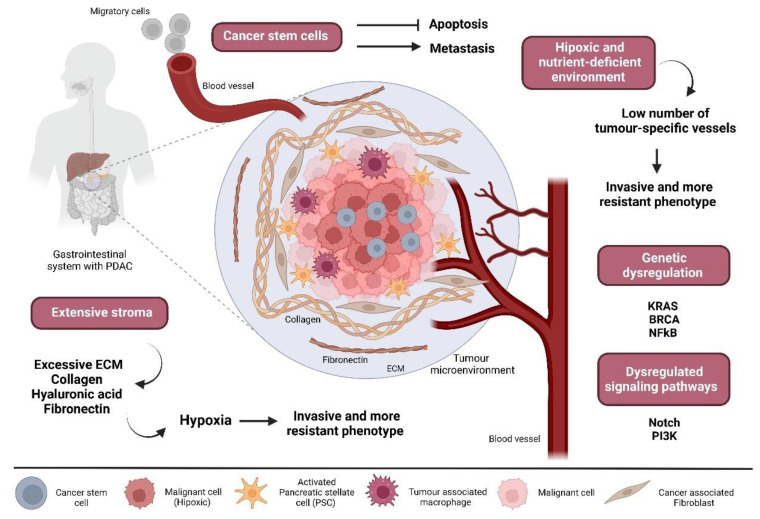
The tumour microenvironment in PDAC. Several cell types are key players in the biology of PDAC. The tumour is characterised by dense desmoplastic stroma that is majorly occupied by PSCs. Tumour-associated macrophages are usually associated with T-cell suppression. CAFs within the PDAC microenvironment are involved in the deposition of the dense ECM typical of the desmoplastic reaction. Dense ECM confers high pressures and solid stress, resulting in vascular compression and reduced diffusion into the tumour that eventually leads to a more invasive and resistant phenotype. Hypoxia drives many signalling pathways involved in aggressiveness and invasion and leads to the acquisition of metastatic properties. Cancer stem cells are well-known for their contribution to chemotherapy resistance and metastasis. Created with BioRender.com (accessed on 31 October 2022).

The problem of resistance to chemical drugs in cancer therapy is complex and several factors may contribute, such as tumour heterogeneity, physical barriers, the immune system, the microenvironment, and the therapeutic pressures induced by antineoplastic drugs (Figure 1) [2,24]. The development of chemoresistance in PC has been associated with the crosstalk between the tumour microenvironment, cancer stem cells (CSCs), and non-coding RNAs. Interestingly, the latest data shows the importance of the tumour phenotype in the development of chemoresistance in the PC, rather than the tumour genotype [24,25].

Different from other solid tumours, PDAC has a peculiar characteristic: the presence of an extensive desmoplastic stroma around neoplastic cells that can occupy ≥80% of the total tumour volume. In the stroma, the excessive extracellular matrix (ECM) acts as a physical barrier between cancer cells and blood vessels, containing a large number of fibrous proteins (i.e., collagen), polysaccharides (i.e., hyaluronan), and glycoproteins (i.e., fibronectin). During PDAC progression and development, the accumulation of these ECM components distorts the normal architecture of pancreatic tissue, inducing an abnormal configuration of blood and lymphatic vessels. This acquired rigidity of the ECM compresses the blood vessels, reducing perfusion and blocking the delivery of drugs and the immune cells from reaching the tumour [26], contributing to treatment failure [27,28]. Understanding tumour-stroma interactions is important to the development of therapies and studies have been conducted to reduce tissue tension and intratumoral pressure by modification of ECM components using chemical or genetic approaches. The aim is to improve tumour blood perfusion and, consequently, increase drug delivery and response to antineoplastic agents [29,30,31,32,33]. Myofibroblast-like cells are another essential component of the PDAC stroma. They are denominated as pancreatic stellate cells (PSCs) and are particularly activated during pancreatic injury or inflammation, expressing high levels of α-smooth muscle actin (α-SMA) and secreting excessive amounts of ECM [34]. Moreover, PSCs are associated with proliferation, maintenance, and chemoresistance in cancer cells [26,27]. In this regard, some studies are particularly looking for drugs capable of blocking this negative impact of PSCs, although the mechanisms of action remain unclear [35]. Targeting the stromal PDAC components could become a possible approach since it increases drug delivery to cancer cells. However, more studies are needed considering the differences in stromal compositions between humans and mouse models.

Cancer stem cells (CSCs) are a current problem in oncology since they have been associated with metastasis and resistance to anticancer treatment in several human cancers. CSCs have specific characteristics, such as the ability to self-renew and a higher expression of anti-apoptotic proteins and drug resistance genes, and share the regulation of signalling pathways, including NOTCH, WNT, or PTEN [36], with normal stem cells. Pancreatic cancer stem cells (PCSCs) are not different from other cancer stem cells, representing less than 1% of all PC cells. In the tumour microenvironment, these cells are in permanent contact with other types of PC cellular and acellular components, and several studies have established the crosstalk between them [36,37,38]. These different components create a tumour niche, a specific hypoxic microenvironment where CSCs can reprogramme their metabolism in a way that increases tumour proliferation and drug resistance, maintaining phenotypic plasticity [39]. Furthermore, CSCs’ chemoresistance has been associated with mutations of drug targets, metabolic inactivation of the drug [36], and altered drug transporter activity [40]. It will be relevant to identify the specific pathways that allow PCSCs to be part of the chemoresistance processes that can be pharmacologically targeted. Other promising approaches are to identify the specific surface markers of PCSCs for antibody-targeted therapies, gene silencing approaches, or even the use of natural products that promote the differentiation of CSCs only into normal tissue cells.

Hypoxia is a well-known problem in many solid tumours, being capable of improving cancer cells’ metabolism to a more resistant phenotype [41]. Hypoxia induces several intracellular signalling pathways, with an active role in cell proliferation, metabolism, apoptosis, invasion, and inflammation [41,42]. Despite the lack of consistent studies, evidence of intra and intertumoral heterogeneity of hypoxia in pancreatic cancers has been reported [43,44], linked specifically to the abovementioned dense stroma [44]. Stromal cells in PDAC increase the production of antiangiogenic substances and contribute to poor levels of O_2_. Angiographic studies of PDAC tissue revealed a low number of tumour-specific vessels, demonstrating that angiogenesis in pancreatic cancers is not effective [45,46]. PCSCs can be activated by hypoxia and secrete more amounts of ECM, perpetuating the vicious cycle of fibrosis and hypoxia [47]. Eventually, PC cells will adapt to hypoxia, promoting an invasive and more resistant phenotype that in most cases does not respond to adjuvant therapies [48]. Experimental evidence confirms the metastatic potential of PC (induced by the endothelial-to-mesenchymal transition (EMT)) with lower O_2_ levels [48,49]. Hypoxia-induced signalling plays a role in most, if not all, of the steps involving metastasis. Therapeutic approaches based on targeting hypoxia can be designed to inhibit the HIF-1 pathway, increasing tumour perfusion or enabling therapies that counteract the metabolic reprogramming of PC due to hypoxia [50,51].

## 3. Marine Organisms and Anticancer Drugs for Pancreatic Cancer

**Figure 2:**
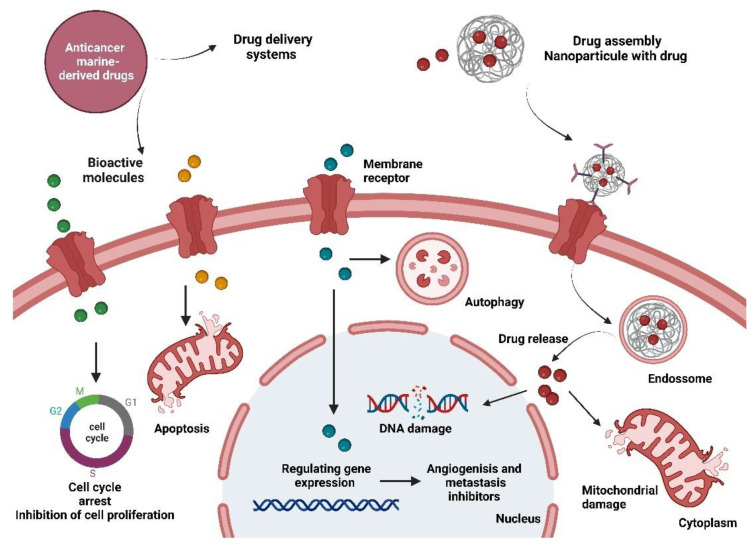
Overview of the anticancer activity of marine-derived compounds for cancer treatment. The main targets of these molecules are the inhibition of cell proliferation and cell viability through cell cycle arrest and DNA damage, induction of ROS production and mitochondrial dysfunction, autophagy, and apoptosis. These drugs can also be administrated by delivery systems that promise control of drug release, targeting ability, enhancement of therapeutic efficacy, and protection of the drug against the immune host system as well as a reduction in toxicity due to high doses in systemic administration. Created with BioRender.com (accessed on 13 October 2022).

Since ancient times, natural products have been explored for their potential therapeutical effects, such as anti-inflammatory, antioxidant, analgesic, and antitumour activities, among others. Natural products are essential for supporting the development of new drugs and there are numerous molecules with antineoplastic activity derived from marine organisms, microorganisms, and plants. Polyphenols, polysaccharides, alkaloids, and peptides are examples of the huge diversity of molecules that are being studied, involving a synchronised effort of multidisciplinary research areas to extract, isolate, and identify compounds to turn them into promising leads [52]. The results of several studies summarised in this review suggest that the compounds derived from marine organisms being evaluated for pancreatic cancer therapies may exert activity by inhibition of cell proliferation and cell viability, induction of ROS production, mitochondrial dysfunction, ER stress, and apoptosis (Figure 2). However, more studies are necessary to more precisely understand the mechanism of action of these molecules in cancer cells, including by considering new cancer models yet to be established.

### 3.1. Marine Sponges

In abyssal deep-sea environments, the most ancient multicellular animals on earth, sponges (a phylum of *Porifera*), contribute to approximately 30% of all marine natural products discovered to date. With a worldwide fauna of at least 15,000 species, they are fundamental to performing studies of animal evolution [3]. Since they do have not an innate immune system or refined defence structures, they persevere by producing metabolites that act as a self-defence device that allows them to adapt to the most diverse environments of the evolutionary scale and hinder predators. Nowadays, the biological potential of these chemicals and metabolites has been extended to biomedical sciences with multiple effects in molecular and cellular events. More than 60 compounds obtained from sea sponges have shown anticancer activities through the induction of apoptosis and/or anti-proliferative effects [52,53,54]. 

For cancer, in general, the most attractive drugs derived from marine sponges are Protein Kinase C (PKC) inhibitors since extreme levels of PKC enzymes are correlated with cancer development [55]. Antitumour activity was found in substances from diverse species of sponges and are described as non-specific inhibitors, well-known because of their toxic effects on healthy cells [56,57], specific inhibitors that can act directly on tumours [58], and inhibitors of specific types of cancer cells [59]. Furthermore, there are already molecules derived from deep-sea sponges that have been clinically approved. For example, cytosine arabinoside (AraC), from the Caribbean sponge *Tethya crypta* [52], and Eribulin, derived from the sponge *Halichondria okadai,* have been used against pre-treated metastatic breast cancer cells [60]. For pancreatic cancer, in particular, there is an increasing demand for deep-sea derived metabolites with antitumour potential. Promising results on the inhibition of Interleukin-8 by Theopederins K and L from the marine sponge *Discodermia* sp. and Mycalamide A from *Mycala* sp. have been reported in the literature. These molecules are non-specific proteins that can inhibit the secretion of interleukin-8 in several PC cell lines [61]. Since interleukins activate survival-signalling pathways and promote metastasis, the ability of these drugs to inhibit Interleukin-8 needs to be fully understood, and more studies are required [62].

An important class of drugs in cancer treatment are anti-proliferative drugs that mainly interact with the cell cycle progression. Aphrocallistin was isolated from the deep-water Hexactinellida sponge *Aphrocallistes beatrix* and shows moderate inhibition of the PANC-1 human pancreatic carcinoma cells’ proliferation with significant cell cycle arrest [62]. Additionally, in the same study, greater inhibition of tumour proliferation was observed for cell lines with a p53 mutation, which occurs in most of the PC types and is associated with poor survival [62,63]. Other interesting compounds, named batzellines, are pyrroloiminoquinones alkaloids, obtained from the deep-water Caribbean sponge *Batzella* sp., and their cytotoxic effects have already been elucidated in murine leukaemia cells [64]. The main mechanism in PC cell lines seems to be the inhibition of topoisomerase II and their capacity to affect DNA synthesis by intercalating into DNA, although other mechanisms may be involved. The results obtained with these alkaloids show higher cytotoxicity to PC cells than 5-fluorouracil (previously mentioned as a current chemotherapeutic drug in PC) [65]. 

Another important class of drugs is the apoptosis inducers and some of them can also be associated with the inflammatory pathway since both are closely related. The cyclic peptide Microsclerodermin A, isolated from the sponge *Amphibleptula*, has previously shown antifungal and anti-proliferative activities in specific cancer cell lines [66,67,68]. Its ability to induce apoptosis was proven in PC cell lines but the mechanism of action is not fully understood. Microsclerodermin A inhibits the nuclear factor kappa B (NFκB) transcriptional activity, reducing the levels of phosphorylated (active) NFκB in the AsPC-1 cell line mediated by the glycogen synthase kinase 3β pathway. The NFκB pathway is continuously activated in PC and its activation is highly associated with metastatic potential and resistance to apoptosis, both found in other cancers and inflammatory diseases [67]. Until now, molecules capable of inhibiting NFκB are not used in the clinic, in part because their mechanism of action is not completely understood, suggesting the need for additional studies. 

To date, the most successful and commercially accessible anticancer drug isolated from marine sponges is eribulin mesylate (EM), however, for PC, only pre-clinical studies were performed [3,60]. EM acts on the abnormal tumour vasculature, increasing the formation of micro vessels and thus increasing tumour perfusion [2,69]. Due to the characteristics of PC, this drug could bring several benefits to patients, such as improving drug delivery and decreasing the levels of hypoxia. The compounds herein reported highlight the great value of marine sponges as a promising source of anticancer molecules for PC treatment. 

### 3.2. Marine Molluscs

Marine molluscs can be found in tropical seas and temperate waters from the Arctic to Antarctic regions, occupying a wide range of ecological niches. These marine organisms have followed the natural course of evolution, producing bioactive molecules adapted to the environmental conditions, mainly to prevent consumption by predators. This ability to adapt to different external stresses has allowed them to synthesise secondary metabolites with immunological and anti-microbial activities [70]. Several compounds derived from marine molluscs such as alkaloids, carotenoids, and conotoxins have been tested over the years in the cancer research field. Nevertheless, there is still much to be explored regarding the discovery of new drugs.

A significant number of compounds derived from molluscs have gained attention as anticancer molecules but, unfortunately for the field of pancreatic cancer, only a few of them have been tested or the tests performed were not conclusive. One of the most known compounds was first isolated from *Dolabella auricularia* and then discovered (with its methyl derivative, symplostatin 1) in the Cyanobacteria *Symploca hydnoides*. It was named Dolastatin 10, a linear pentapeptide that inhibits the microtubule assembly, eventually leading to metaphase arrest in the cell cycle. Unfortunately, it did not pass clinical trials for different types of cancers, including PDAC, because of the severe side effects and the ineffectiveness of the drug in safe doses [71]. Another mollusc-derived compound is Elisidepsin (PM02734, Irvalec^®^), a synthetic cyclic peptide of the Kahalalide F family, currently in clinical trials (phase II). Elisidepsin induces the downregulation (dephosphorylation) of ErbB3 protein in PC cell lines. Its combination with other chemotherapeutics or ErbB-targeted drugs can be a viable option to improve the efficacy of cancer treatment. In addition, in vitro studies have reported that elisidepsin induces a rapid loss of membrane integrity in cancer cells, accompanied by a significant Ca^2+^ influx and perturbations of membrane conductivity [72]. The potential of this compound as a biomarker for resistant cancer cells also needs to be explored once it is more active in cells with high E-cadherin and low vimentin, both linked with cancer cell evasion and metastasis in PC [72,73]. 

The bioactive metabolites derived from molluscs are only available in small amounts. However, preclinical evaluation, including in vivo studies with rodent models, requires higher amounts of the pure compound, a quantity that will need to increase substantially in clinical trials. The problem of supply is a constant concern that researchers need to overcome. In recent years, new techniques in harvest/culture and isolation processes, the increased knowledge of genomics, as well as advances in chemical synthesis, have offered great promise for the large-scale production of metabolites derived from marine molluscs [70]. 

### 3.3. Bryozoans

Bryozoa (sea mats, moss animals, or lace corals), a phylum of filter-feeding invertebrates, are abundant and important members of several benthic communities in a variety of marine habitats. They are sources of pharmacologically interesting molecules, including alkaloids and polyketides, with unique structural and bioactive diversity [74]. 

In the alkaloid group, Amathaspiramides A–F were isolated from *Amathia wilsoni* Kirkpatrick, 1888 (Vesiculariidae) by Morris and Prinsep [75]. These compounds were tested in vitro for antiproliferative activity and only Amathaspiramide C exhibited promising results against a PC cell line (MiaPaCa-2). This study highlights the importance of the amine or imine substructure on the pyrrolidine moiety and the 8R stereochemistry on the N-acyl hemiaminal moiety for the antiproliferative activity of amathaspiramide alkaloids [76]. 

Currently, the most promising class of marine-derived compounds from bryozoans in cancer drug development is Bryostatins. Their low toxicity and antineoplastic activity both in vitro and in vivo have attracted the attention of researchers. Bryostatins can selectively modulate the function of diverse PKC enzymes, which represent a key role in the regulation of cell growth and death [74]. They are also the most explored class in PC, specially Bryostatin-1, with preclinical and clinical studies as mono or combined therapies. Alone, Bryostatin-1 is capable of substantially reducing tumour growth in vivo through the downregulation of NF-κB expression and inducing apoptosis in vitro in pancreatic carcinoma cells (MiaPaCa-2) [77]. In combination, Bryostatin-1 was tested with Paclitaxel, but it was an unsuccessful therapy for patients with advanced pancreatic carcinoma [78]. In contrast, the combination of Bryostatin-1 with GEM in a phase I clinical trial seemed to be well tolerated with promising antitumour activity. However, PC patients were not involved in this study, highlighting the importance of phase II clinical trials including these patients [79,80]. 

This phylum has received little attention until now, mainly because of the insufficient biomass of bryozoan samples. Many species are deeply calcified, making the collection process more difficult. For this reason, the isolation of secondary metabolites has been negatively affected. A possible option could be synthesising analogues of these compounds in the laboratory. The syntheses of six bryostatins (1, 2, 3, 7, 9, and 16), retaining their biological activity and consistent in vitro antitumour effects, even with their simplified structure, have already been published [81]. More efforts are needed to collect or synthesise bryozoan analogues that will eventually lead to products with high biomedical potential. 

### 3.4. Macroalgae

Macroalgae, also known as seaweeds, are photosynthetic organisms that can be divided into three different groups: brown algae (Ochrophyta, Phaeophyceae), green algae (Chlorophyta), and red algae (Rhodophyta) [82]. Seaweeds are rich in lipids, minerals, and certain vitamins, and are one of the major producers of several bioactive agents such as polysaccharides, proteins, and polyphenols. They present attractive biological and pharmacological effects on cancer, diabetes, and inflammation, likely leading to significant progress in the biomedical field [83,84,85,86]. These bioactive compounds may be used as new chemotherapeutic drugs or as building blocks for anti-cancer systems, including drug delivery devices. Recent findings demonstrate the anti-proliferative, anti-angiogenic, pro-apoptotic, and cell cycle arrest of these seaweed compounds for different types of cancers such as melanoma and lung, breast, and colon cancer [87,88,89,90]. However, few studies regarding pancreatic cancer have been reported. In the section below, fucoidan and polyphenols will be particularly addressed as examples of bioactive agents that can be extracted from seaweeds. 

#### 3.4.1. Fucoidan

Fucoidan, a sulphated polysaccharide extracted from brown algae, may present different biological activities such as antitumour, anti-inflammatory, antioxidant, and anti-angiogenic effects [91,92,93]. Its use in the cancer field has been extensively studied over the last few years [94]. Fucoidan extract from *Turbinaria conoides* (Phaeophyceae) was studied in distinctive pancreatic cancer cell lines, inhibiting cell proliferation in a dose and time-dependent manner [95]. In addition, fucoidan induced caspase −3, −8, and −9 activation, indicating cancer cell apoptosis. Moreover, fucoidan increased cellular p53, which is mutated in 50% of pancreatic cancers, suggesting a targeting approach that may induce cell apoptosis. In a different study, the anti-cancer effects of fucoidan extracted from the same species were investigated [96]. Fucoidan inhibited cell proliferation and induced apoptosis in pancreatic cancer cell lines. Its antioxidant and anti-angiogenic potential were also assessed, showing efficacy against different free radicals and a reduction in the number of blood vessels, both in vitro and in vivo. These results indicate that fucoidan is a promising marine-origin polysaccharide for the development of pancreatic cancer treatment strategies that should be further explored.

#### 3.4.2. Polyphenols

Marine macroalgae are rich in polyphenolic compounds that can be classified into different categories depending on their source, biological function, and chemical structure [97]. Polyphenols can be divided into phenolic acids, flavonoids, stilbenes, lignans, and other phenolic compounds. Their consumption has been linked with a reduced risk of some diseases, such as cancer, cardiovascular, metabolic, and neurodegenerative disorders [98,99,100]. Regarding cancer, polyphenols showed antiproliferative activity against colon and breast tumours [101]. Similarly, a polyphenol from a different extract showed apoptosis-enhancing effects on the MCF-7 human breast cancer cell line [102]. Furthermore, crude extracts from brown algae exhibited cytotoxicity against the cervical cancer HeLa cell line [103]. The anticancer mechanism of polyphenols has yet to be established; inhibiting the formation of mitotic spindles by preventing normal microtubule formation has been suggested as one possible mechanism [104].

The effect of polyphenols extracted from different brown algae was also studied for pancreatic cancer [105]. The effects on different tumour progression molecular targets, the inhibition of cell proliferation, and the induction of pancreatic cancer cell apoptosis were explored. These compounds presented selective cytotoxicity since the proliferation of normal cells was not affected, which is a parameter of the utmost importance when considering effective antitumour therapies. In a different approach, the authors studied the ability of three different polyphenols with anti-pancreatic behaviour to target the main signalling pathways involved in activating and/or maintaining cancer stem cells after first-line therapy [106]. The polyphenols inhibited pancreatic cell proliferation in a dose-dependent manner. Results showed that seaweed polyphenols inhibited the recurrence of pancreatic cancer by targeting therapy-orchestrated stem-cell signalling in residual cells. In their latest publication, the same authors studied the effect of one of the previously studied polyphenols in regulating the dissemination of therapy-resistant PC cells in vitro and residual PC in vivo [107]. The seaweed polyphenol specifically targets the radiation-induced CXCR4-/COX-2-dependent dissemination destiny of surviving PC cells, being a promising and potential drug for PC treatment. In another attempt, the authors investigated if polyphenols would be able to regulate autophagy, since targeting this process is essential to finding a cure for pancreatic cancer (activated autophagy is associated with poor patient outcomes) [108]. Results demonstrated that polyphenols presented increased cytotoxicity in therapy-resistant pancreatic cancer cells. In addition, these polyphenols prevented and targeted radiotherapy-activated autophagy, suppressing the transcription of studied autophagy regulators in both cell lines. In a different study, three seaweed polyphenol drug candidates (SW-PD) were investigated [109]. Treatment with the SW-PD led to the mitigation of oncogenic burden and repressed critical drivers of tumour genesis, dissemination, and evolution. This was validated by the increased cell death of resilient pancreatic cancer cells. In addition, this marine-based treatment impaired the increased localisation of oncoproteins in residual tumours after radiation therapy. These seaweed polyphenols may be a promising therapeutic approach in combination with other treatments. Despite the promising results regarding seaweed polyphenols and their anti-cancer potential, their mechanism of action is still unclear, which is why more comprehensive studies need to be performed. 

### 3.5. Marine Fungi

Fungi are a group of eukaryotic organisms that obtain their nutrition by absorbing and breaking down organic matter. The fungi kingdom is present in almost every ecological niche, yet they represent a less explored source of bioactive molecules. It is estimated that only 10% of all species are known by the scientific community. Their pharmacological potential has gained special interest since the discovery of penicillin and currently, several compounds are reported with a vast number of biological effects such as anticancer, antioxidant, immunoregulatory, hepatoprotective, antibacterial, antidiabetic activities, etc. [110]. Although this review addresses only their pharmaceutical applications, particularly on pancreatic cancer therapeutics, fungi also are promising sources of enzymes with technological applications in environmental, textile, biofuel, and agriculture fields well-reviewed by Bonugli-Santos [111]. 

When we direct our view to the marine ecosystem, their potential exponentially increases. Marine fungi can be found in different substrates such as sediments, algae, sponges, and molluscs, among others [112]. Like other marine organisms, they are exposed to different environmental conditions than terrestrial fungi, which directly contribute to the differences between the enzymes and metabolites generated. They are producers of high- and low-molecular-weight bioactive compounds (alkaloids, lipids, peptidoglycans, phenolics, polysaccharides, proteins, polysaccharide-protein/peptides, ribosomal and non-ribosomal peptides, steroids, etc.) owning more than 130 different therapeutic effects [113]. In cancer research, they can be alternatives for cytotoxic and antiproliferative agents in several types of cancer [114,115]. 

The most promising results obtained for PC come from the cyclodepsipeptides, scopularides A and B, found in the fungus *Scopulariopsis brevicaulis*, which was isolated from the marine sponge *Tethya aurantium*. Both metabolites have demonstrated specific activities against tumour cell lines, including PC cells (Colo357, Panc89). Treatment with scopularides A led to a reduction in PC cell line viability by 36%, whereas scopularides B reduced the viability of the PC cell line by 26%. The authors concluded that scopularides A can be more toxic than scopularides B in the PC cell line (Panc89) [116]. Another cyclic peptide, clavatustide, was isolated from the metabolites of mycelia cultivated with the hydrothermal fungus *Aspergillus clavatus*. Clavatustide B is an effective anti-proliferative drug in colorectal cancer (SW-480), retinoblastoma (WERI-Rb-1), prostate cancer (PC3), and human pancreatic cancer (Panc-1). Its mechanism of action appears to be linked with the regulation of the G1-S transition of the cell cycle [117]. Despite these results, these molecules need further investigation to determine their mechanism of action, mainly in PC cells. In the same way, sansalvamide A, produced by a fungus of the genus *Fusarium* living on the marine plant *Halodule wrightii,* inhibits topoisomerase I in PC cell lines (AsPC-1 and CD18), with a significant decrease in cell proliferation [118]. Other studies in PC cells with analogues, reported the G0/G1 phase cell cycle arrest and the decrease in protein expression related to the cell cycle (cyclin E and cdk2). The number of apoptotic cells also increased in the studied PC cell lines [119]. These studies reported the anticancer activity of cyclic peptides derived from marine fungi in several types of PC cells, but they need additional studies in cancer animal models to better understand their in vivo behaviour.

More recently, Zhang et al. found that libertellenone-H isolated from Arctic marine fungi inhibits the thioredoxin system, triggering potent ROS-mediated apoptosis in human PC cell lines. This study offered significant information for the clarification of the anticancer activity of this pimarane diterpene and the development of a new type of molecule targeting the thioredoxin system with high specificity and activity against cancer cells [120]. It is important to note that the thioredoxin system is an important antioxidant system in defending against oxidative stress and regulating cellular redox homeostasis by eliminating ROS. This system plays a key role in tumour initiation, progression, and drug resistance and seems to be overexpressed in many human cancer cell lines and human tumours, including PC [121,122]. For this reason, molecules targeting it can be important for cancer research. 

The marine-derived fungus *Aspergillus terreus* has been a source of new promising molecules for cancer therapy, such as lignans, meroterpenoids, and alkaloids. Changxing Qi et al. tested twelve butenolide derivatives, including three new compounds, asperlides A–C, and nine known analogues isolated from this fungus. The results showed that two of the compounds, (+)-3′,3′-di-(di-methylallyl)-butyrolactone II and versicolactone B, exhibited the most potent cytotoxic activity in the PANC-1 cell line. Additionally, (+)-3′,3′-di-(di-methylallyl)-butyrolactone II inhibited the proliferation of PANC-1 cells via the induction of G2/M and S phase arrest, while versicolactone B retarded the PANC-1 cells via the induction of S phase arrest. Flow cytometric analysis suggested that treatment with these two compounds significantly induced apoptosis in PC cells [123]. 

These findings suggest that compounds derived from marine fungi might serve as a starting point for the development of an anticancer drug for the treatment of PC. However, due to the limitations of the in vitro studies, more research should be performed to clearly understand their mechanisms of action, namely by using animal models and more complex in vitro cancer models.

### 3.6. Marine Bacteria

The ocean’s water column comprises nearly 10^6^ bacterial cells per millilitre. Some of these species live in low temperatures, high pressures, darkness, or/and low-oxygen conditions that force them to adapt to these extreme environments. Surviving in these conditions makes them able to develop unique metabolic and physiologic systems producing biomolecules with fascinating complexity and diversity. Unfortunately, unlike their terrestrial counterparts, to the best of our knowledge, no drugs isolated from marine bacteria have been approved by the FDA, but the number of new molecules that are currently in evaluation is optimistic [124].

Cyanobacteria and Actinobacteria-derived compounds are so far the most described, followed by Proteobacteria, Firmicutes, and Bacteroidetes. Indeed, the most promising molecules described until now are derived from Proteobacteria and filamentous Cyanobacteria [124,125]. Marine bacteria-derived compounds have demonstrated antioxidant, antibacterial, apoptotic, antitumour, and antiviral activities with potential therapeutic value [126,127]. Special attention has been given to their antimicrobial activities because of the increasing concerns about bacterial resistance to conventional antibiotics [128]. More than that, antiproliferative, cytotoxic, and antimetastatic activities against cancer cell lines have also raised the curiosity of scientists to find new molecules to treat human cancers. Several compounds, including Apratoxins, Cryptophycins, Largazole, Proximicin, and others, are currently in pre-clinical tests aiming for potential therapeutic uses in oncology [129,130,131,132]. Unfortunately, currently, only a few of these drugs have been tested for pancreatic cancer therapy and the available studies are insufficient to predict future outcomes. However, there are a few marine bacterial-derived molecules that were already tested in pancreatic cancer cell lines that allow us to understand their performance in this cancer. 

Apratoxins are a class of cyclodepsipeptides isolated from marine cyanobacteria. Type A has shown the most potent cytotoxic activity in different cancer cell lines, including breast, ovarian, and colon, but with high in vivo toxicity, which limited its therapeutic use [129,133,134]. Apratoxin A induces G1 arrest and antagonises the FGFR signalling pathway by inhibiting the phosphorylation of STAT3 [135]. Experiments performed using cancer mouse models also support these findings [136]. Other studies report the ability of Apratoxins to act as anti-angiogenic agents, downregulating both receptor tyrosine kinases (RTKs) and their ligands including VEGF-A and IL-6 [137,138]. Cai et al. hypothesised that Apratoxins have a high affinity to pancreatic cells since the secretory machinery in this organ is the natural target of Apratoxins, leading to deeper investigations related to PC. In fact, these authors reported the growth inhibition of PC cells and stromal cells by the downregulation of several receptor tyrosine kinases, inhibition of growth factors, and cytokine secretion. However, due to the toxicity shown by Apratoxin A, an analogue was synthetised, Apratoxin S10 (Apra S10), with more stability, potency, and production yield [139]. These molecules need a deeper investigation to be a useful adjunct to current cytotoxic therapies for PC. Another potential compound is Coibamide A, also isolated from Cyanobacteria, which demonstrated antiproliferative activity against human cancer cells and an exceptional selectivity profile. Coibamide A inhibits VEGFR-2 and its ligand VEGF-A and induces mTOR-independent autophagy [140]. Its action mechanisms appear to be very similar to Apratoxins and, for this reason, it may be interesting to test this compound against PC cells.

Marine bacterial exopolysaccharides have been isolated from water columns, sediments, or animals, and have gained increasing attention as sources of potential new cancer drugs [141]. Exopolysaccharide 11 (EPS11), isolated from the marine bacterium *Bacillus* sp. 11, displayed anti-tumour and antimetastatic activities in vitro and in vivo in liver cancer. Different from the other compounds, EPS 11 targets type I collagen and inhibit its synthesis via the β1-integrin signalling pathway. β1-Integrin acts as a transmembrane receptor and plays a crucial role in cell–ECM interactions [142]. The migratory and invasive phenotype of cancer cells can be inhibited by anti-β1-integrin monoclonal antibodies in pancreatic carcinoma [143]. In the same way, the knockdown of β1-integrin significantly decreases primary tumour growth and inhibits PC metastasis [144]. Although EPS 11 bioactivities were not explored in PC cell lines or models, it appears that it can be tested to target the PC stroma and ECM that contains excessive amounts of collagen and other ECM proteins. The mechanism of antitumour action of exopolysaccharides is related to the stimulation of the immune system, triggering of cell apoptosis, and activation of autophagy, but so far, no reports about the exact targets of the antitumour effects of exopolysaccharides are available [145].

Marine bacteria are promising microorganisms for the isolation of novel molecules with anticancer properties, also envisaging the possibility of producing them in bioreactors and thus establishing a sustainable production system. However, a lot of information is lacking, mainly in the field of pancreatic cancers, which adds to the bottleneck that may be associated with the cultivation of some marine bacteria.

### 3.7. Clinical Trials with Marine Natural Products Addressing Pancreatic Cancer

Many novel therapeutic strategies are currently under investigation. They comprise a broad range of therapies targeting different biologic processes. However, few novel therapies are currently in late-stage testing, suggesting that future progress is likely several years away. Table 1 shows the results of several clinical trials investigating the potential of several marine-derived compounds (as single agents or in combination with other standard or conventional drugs). A few clinical trials are testing the efficacy of marine-derived compounds, but some of them can be promising candidates to treat PC or at least be integrated into combined therapies. For this reason, understanding the mechanisms of action of these drugs in pre-clinical studies is essential to select the best candidates. 

## 4. Marine-Inspired Materials for Drug Delivery in Pancreatic Cancer

Current cancer therapies are mainly surgery, radiotherapy, and chemotherapy that can be used in a single or combined way. Chemotherapy is an adjuvant for patients that undergo pancreatic resection as the main therapy for incurable pancreatic cancer [154]. Most chemotherapeutic drugs present limited bioavailability and poor water solubility, requiring increased drug dosages to achieve therapeutic effects. The nonspecific biodistribution of anti-cancer agents results in adverse and unwanted side effects, particularly cytotoxic effects over normal healthy tissues [155]. In addition, there are problems associated with the low drug penetration index due to the complexity of the tumour microenvironment, the development of drug resistance, the degradation, and the short half-life of chemotherapeutic drugs [156]. Some of these limitations may be surpassed by drug delivery systems (DDS), widely used to develop more effective anti-cancer therapies. Most DDS being developed are different nanoparticles (NPs) capable of successfully delivering chemotherapeutic drugs to their site of action with diminished side effects [157]. This happens since tumours present a leaky vascularisation leading to the accumulation of the NPs at the desired sites [158]. NPs can be tailored with different targeting motifs (for example, antibodies) that can be recognised by cancer cells [159]. These systems can increase the circulation time of the drugs and protect them from enzymatic degradation, which will reduce the need for increased dosages [160]. NPs based on natural polymers may be promising DDS due to their biocompatibility and biodegradability, namely, for their application in pancreatic cancer [154]. 

In this section, marine-based DDS will be presented and discussed as different strategies for pancreatic cancer treatment. Chitosan (produced from crustacean shells or squid pens), the most common marine polysaccharide used for the development of DDS in pancreatic cancer, along with fucoidan and alginate (extracted from brown algae) will be given as illustrative examples. Table 2 summarises the main findings related to these systems.

### 4.1. Chitosan-Based Drug Delivery Systems

GEM is a commonly used chemotherapeutic agent for patients with advanced pancreatic cancer, while EGFR (epidermal growth factor receptor 1) overexpression leads to increased cell proliferation, being a possible targeting receptor [163,172]. A chitosan-based approach conjugating GEM and EGFR antibodies was developed to deliver biological agents specifically to cancer cells [173]. The developed system encapsulated around 92% of the drug, releasing 87% of GEM in 24 h. The NPs with the antibody showed increased cellular uptake in human pancreatic cancer cell lines (SW1990), proving the targeting efficiency. Additionally, cell proliferation, migration, and invasion were considerably affected. A different antibody was used to prepare Herceptin-conjugated GEM-loaded chitosan nanoparticles (HER2-GEM-CS-NPs) to target pancreatic cancer cells [162]. After optimisation, the nanoparticles’ final formulation resulted in NPs with 300 nm in size and a positive zeta potential of around 18 mV. Approximately 35% of the drug was released within the first 24 h. Cellular binding studies demonstrated the targeting capability of the immobilised antibody, showing an increased fluorescence signalling when compared with unconjugated NPs and HER2 negative cells used as control. In vitro studies showed higher cytotoxicity of the developed NPs than unconjugated NPs and the free drug over two different pancreatic cancer cell lines; HER2-GEM-CS-NPs arrested the cell cycle at the S-phase.

In a different study, from all the pancreatic cancer cells tested, the folate receptor was higher expressed in COLO357, while on normal cells, the expression was significantly lower [164]. Aiming to target this receptor, folate-chitosan-GEM nanoparticles were developed. When folate was incorporated into the NPs, a stronger green fluorescence was observed, as well as significant cytotoxicity over COLO357 cells. In vivo studies showed inhibition of tumour growth when compared with free GEM. This same drug was encapsulated in chitosan/glyceryl monooleate nanoparticles with an average diameter of 380 nm, a positive zeta potential of 22 mV, and a maximum of 40% of GEM released in 48 h [161]. The percentage of accumulated drug and cytotoxic effects increased with GEM-loaded NPs when compared with the free drug. 

Metformin, an FDA-approved drug commonly used to treat type 2 diabetes, also presents effects on cancer cells, with different mechanisms being described. The drug was encapsulated in O-carboxymethyl chitosan NPs and its effects targeting pancreatic cancer therapy were reported [165]. The developed NPs impaired its clonogenic ability, reducing the ability of cancer cells to form colonies. qRT-PCR results showed reduced gene expression, suggesting the importance of the NPs in cell cycle progression. The biodistribution of the developed system was assessed in vivo and no adverse toxic effects were observed, indicating it as a possible and promising approach for pancreatic cancer treatment.

A different group developed chitosan solid-lipid nanoparticles (SLNs) for the delivery of different drugs (aspirin and curcumin) and free sulforaphane [166]. The developed NPs present a size range of 360–440 nm and encapsulation efficiencies of 65–72%, depending on the drug. In vivo studies were performed to assess the toxic effects of combined SLNs chemo-preventive treatment. No changes in blood counts and chemistry were observed and the histopathological analysis of the different organs presented no abnormalities. Taken together, these results, and a previous study from the same group, make these systems a safe strategy for pancreatic cancer prevention.

Chitosan NPs loaded with quercetin and 5-fluorouracil were prepared with a size of around 300 nm when single drugs were encapsulated and 400 nm when both drugs were loaded [167]. NPs were successfully internalised by pancreatic cancer cells in a time-dependent fashion. Quercetin chitosan NPs presented a cytotoxicity around 27% and 45% for 5-fluorouracil chitosan NPs. However, the most effective treatment modality was the combination of both NPs, with a cytotoxicity of around 70% that should be further studied.

Curcumin-loaded Poly d,l-lactide-*co*-glycolide (PLGA) NPs were prepared and further coated with chitosan and PEG to improve the bioavailability and circulation time of the drug, limitations that are often associated with chemotherapeutic drugs [168]. The produced NPs presented an average diameter of 264 nm, a PDI of 0.181, and a positive zeta potential of 19.1 mV. The cellular uptake of the NPs increased when compared with the uptake of the free drug. Curcumin alone presents an IC_50_ of 28 µM and 20 µM for PANC-1 and Mia Paca-2, respectively. The developed NPs showed an increased cytotoxicity compared to the free drug, with IC_50_ values of 14 µM (PANC-1) and 6 µM (Mia Paca-2), respectively. Enhanced anti-migratory, anti-invasive, and apoptosis-inducing capabilities of NPs were also observed.

### 4.2. Fucoidan-based Drug Delivery Systems

Regarding fucoidan usage for the development of DDS, only a couple of studies have been reported so far. Fucoidan-coated manganese dioxide nanoparticles (Fuco-MnO_2_-NPs) were successfully synthesised, resulting in NPs around 50 nm in size and a negative zeta potential of −25 mV [169]. Cytotoxic effects of the developed NPs were assessed over two different human pancreatic cancer cell lines (AsPC-1 and BxPC-3), showing a decrease in cell viability of about 80% for 20 µg/mL. NPs generated oxygen efficiently in the presence of H_2_O_2_ and considerably suppressed HIF-1 expression under a hypoxic condition, reversing the hypoxia-induced radio resistance by increasing DNA damage and apoptotic cell death in response to radiotherapy. The effects of the combination of NPs and radiotherapy were analysed in nude mice bearing BxPC3 xenograft tumours, showing an increased impairment of tumour growth than with radiotherapy alone. Taking advantage of the electrostatic interactions between fucoidan (negatively charged) and lactoferrin (positively charged), NPs were prepared by polyelectrolyte complexation [174]. After optimising different parameters, NPs presented a size of 167 nm, a polydispersive index of 0.197, and a negative zeta-potential of −27 mV. The cytotoxicity of the developed NPs was assessed over the PANC-1 cell line, reducing cells’ viability more than fucoidan alone. The authors attributed this to an easier cellular uptake of NPs. Furthermore, the developed NPs increased the ability to prevent pancreatic cancer cells’ migration and invasion.

### 4.3. Alginate-based Drug Delivery Systems

Photosensitiser-encapsulated amphiphilic sodium alginate derivative (Photosan-CSAD) nanoparticles were prepared to enhance the phototoxicity in the photodynamic therapy of pancreatic cancer [170]. The developed NPs had a size ranging from 150 to 250 nm, a spherical shape, and negative zeta potential of −21 mV. Photosan-CSAD was incubated with human pancreatic cancer cells, showing increased fluorescence activity and reactive oxygen species (ROS) generation, resulting in stronger phototoxicity. Apoptosis was suggested to play a pivotal role in cell death. These results indicate that the developed NPs may be a promising approach for the photodynamic therapy of pancreatic cancer. 

Although most of the DDS involved the use of NPs, in an alternative strategy, GEM was incorporated into spun fibres produced from alginate or chitosan, aiming for a localised drug delivery strategy [171]. Drug encapsulation ranged from 13–52%, being the highest encapsulation efficiency related to the highest polymers’ concentrations. MIA-PaCa-2 and PANC-1 human pancreatic cells seeded on top of the GEM-eluting fibres presented a decreased cell viability compared with bare fibres. A decrease in the spheroid size was observed by applying the developed system in a 3D model.

All these studies and respective findings report promising and potential DDS that may be of great value for pancreatic cancer treatment. Nevertheless, further assays and studies should be performed to understand and further validate these systems and the mechanisms involved, envisioning their application in clinics.

## 5. Looking Forward: The Future of Marine-Inspired Drugs and Biomaterials against Pancreatic Cancer

### 5.1. Biomarkers and Precise Medicine

Remarkable research is being performed in the field of PC; however, even with all the recent advances, the survival rates are still not optimistic. The poor prognosis is attributed to late diagnosis but as of today, there are no biomarkers approved for early diagnosis. Biomarkers are crucial in all stages of disease follow-up, diagnosis, prognosis, and evaluation of treatment responses. There are two main types of biomarkers: prognostic and predictive. The first, associated with the clinical outcomes, is used to identify patients with a more aggressive disease course. Predictive biomarkers measure the likelihood of response or lack of response to a particular therapy, allowing the identification of patients most likely to benefit from a given treatment, thus sparing other patients from the toxicities of ineffective therapies. The development of specific PC biomarkers, mainly in the early stages of the disease, will be a hope for early diagnosis [175]. 

Even with the recent outcomes of marine-derived compounds for cancer treatment, their clinical efficacy in early human clinical trials remains limited. Natural compounds, in general, have not yet benefited from the recent advances in the field of predictive biomarkers compared to conventional therapies. Identifying predictive biomarkers will allow for the selection of PC patients who will possibly respond to these natural-derived therapeutics. Adverse drug reactions and heterogenicity between cancer and patients limit the clinical efficacy of drugs. To overcome these obstacles, the development of predictive biomarkers will be particularly advantageous to guide oncologists to choose the most efficient treatment for patients, reducing toxicity [176,177]. 

The current advances in cancer treatment personalisation make it clear that novel therapeutic compounds are urgently needed to improve the drugs’ availability for specific molecular targets. No evidence-based personalised treatment for pancreatic cancers is currently available for clinical practice. The sea already provides a huge number of molecules with therapeutic potential, and novel molecules are yet to be explored.

### 5.2. Three-dimensional Cancer Models

Only a few oncological drugs in clinical development that enter phase I achieve approval. Inappropriate biodistribution and off-target toxicities are common reasons for the unsuccessful results in patients. Currently, in preclinical studies of a PC 2D cell culture, animal and xenograft approaches are used. However, these strategies fail to reproduce the tumour microenvironment and its molecular components precisely, leading in part to non-translatable results. Various preclinical PDAC models have been developed, and each approach has contributed to important aspects of the investigation of PDAC pathogenesis. However, developing new model systems is a current challenge in research [178]. Reproducing tumour complexity does not exclusively require malignant cells, but it must reproduce the microenvironment that constricts or nurtures the tumour mass.

Marine-derived biomaterials have also been explored for clinical applications in dentistry, oral and maxillofacial surgery, cartilage, and bone tissue engineering. Their diversity yields tremendous potential, offering various organisms from which promising natural substances can be isolated to mimic the tissue ECM in the body [179,180,181]. Collagen, for example, is a structural component of ECM in all connective and interstitial tissue. Many non-mammalian species, both vertebrate and invertebrate, have been evaluated as new and alternative collagen sources for tissue engineering. Although marine-derived biomaterials are extensively studied for tissue engineering, namely regarding cell regeneration (bone or cartilage, for example), their potential for developing cancer 3D models has not yet been deeply explored. Sustainable, marine-derived biomaterials are promising candidates as an alternative to mammalian/vertebrate sources, reducing the impact of using mammalian species and their derived products [182]. 

No studies using marine materials for pancreatic cancer 3D models have been reported. However, marine-derived collagen was used to mimic ovarian cancer, supporting cells’ proliferation and expression of epithelial mesenchymal transition markers. In collagen 3D scaffolds, ovarian cancer cells migrated and differentiated, highlighting its suitability for advanced cancer cell culturing applications [183]. Three-dimensional models offer the potential to mimic the dense stroma associated with tumour microenvironments in PDAC, providing a physiologically relevant tool for biomedical research and preclinical drug testing. Collagens are abundant in the ECM of PDAC, with several implications on tumour biology. Developing fibrous scaffolds to mimic the matrix composition and architecture of primary tumour tissues would provide a better understanding of cancer biology and drug delivery. For these reasons, 3D cell cultures using collagen gels have been extensively required to study cell-matrix interactions in PDAC [184]. Thus, testing the marine-originating collagen in 3D models of PDAC may be a viable option with promising results. 

Using marine-derived biomaterials in 3D cancer models will address the global drive for technological developments that result in the replacement of animals and their derived products in research. However, the use of marine species or their derivatives must always consider the need for the conservation and sustainable use of marine biodiversity.

### 5.3. Immunotherapy 

Cancer immunotherapy is currently a promising anti-cancer treatment with several drugs already approved by the FDA and several others in clinical trials. Despite the remarkable efforts made until now, it is still limited to several types of tumours that are sensitive to the immune system (e.g., prostate cancer, urothelial carcinoma, and metastatic melanoma). Approximately 50% of the cell mass of PDAC are immune cells that directly suppress the host immune system and contribute to treatment resistance [185]. No standard treatment based on immunotherapy is approved for PC and, for now, there are only a few possible future treatments in clinical trials [186,187]. Several characteristics of PC can be targeted, including several components of the PC microenvironment (fibroblast, microphages, regulatory T-cells, etc).

Marine-derived products can effectively enhance the therapeutic effects of pre-existing immunotherapies. Cancer vaccines, immune-check points inhibitors antibodies, and adoptive cell immunotherapy are possible strategies available. Marine-derived products can also directly act as immunomodulators, downregulating the secretion of immunosuppressive factors (TGF-β, IL-10), upregulating the secretion of immune factors (IFN-γ and TNF-α), downregulating immunosuppressive cells (Regulatory T-cells and macrophages) and enhancing the number of effector T-cells [185,188]. 

To increase the number of available drugs to target PC, more data should be produced and gathered to provide information about the specific mechanisms of action of marine-derived compounds, not only directly over tumour cells [189], but also in the immune cells of this cancer. Immunotherapy may be enhanced using drug delivery systems that can selectively target immune cells and improve outcomes. As we previously discussed, marine biodiversity offers different strategies in this line that could provide promising molecules against PC and further investigations are most needed to explore this route.

## 6. Conclusions

PC remains an incurable disease in most cases and the need for new therapies is a priority. Marine-derived compounds offer a huge platform for new drug discovery, but multidisciplinary teams are needed to access the sea´s biodiversity to identify the novel compounds and explore their potential biological activity. The challenge these days is how to access this natural chemical diversity. 

Personalising each patient‘s treatment based on their tumour biology is a current goal in clinical oncology, delivering the right treatment to the right patient. Together with personalised chemotherapy, the ultimate therapeutic goal is to use specific drug delivery systems to enable the adequate action of the drug, with some of the routes explored so far being based on materials with a marine origin, as herein discussed.

The development of specific biomarkers for monitoring treatments is at the same time a challenge in PC. The increase in new drug availability, early diagnostic and prognostic methods, and novel models in cancer research will undoubtedly have a positive impact on the survival rates of one of the deadliest cancers. 

## Figures and Tables

**Table 1:** Clinical trials of marine-derived drugs for pancreatic cancer treatment.

Tested Drugs	Phase Trial	PC Type	Main Outcomes	Ref.
Cisplatin, cytarabine, caffeine, and fluorouracil	II	Advanced PDAC	Produced significant responses in PC but the toxicity was significant	[146]
Fluorouracil Plus Folinic Acid vs. Gemcitabine	III	Resected PDAC	Compared with the use of fluorouracil plus folinic acid, gemcitabine did not result in improved overall survival in patients with totally resected PC	[147]
Eribulin mesylat	II	Gemcitabine refractory PDAC	The drug was well tolerated and did not result in any responses in refractory PC	[148]
Paclitaxel plus Bryostatin-1	II	Locally advanced or metastatic PDAC	The combination of these drugs was not an effective therapy	[78]
Trabectedin	II	Gemcitabine refractory and Metastatic PDAC	Some ability to modulate inflammatory process was reported but single-agent trabectedin had no activity as salvage therapy	[149]
Lurbinectedin	II	Advanced PC with DNA repair mutations	Ongoing	[150]
Marizomib and vorinostat	I	Metastatic PDAC	The combination of the full dose was tolerable in patients, with safety findings consistent with either drug alone	[151]
Hydroxychloroquine in combination with gemcitabine and nab-paclitaxel	II	Resettable PDAC	Greater tumour response, improved serum biomarker response, and evidence of autophagy inhibition and immune activity were reported	[152]
Nab-Paclitaxel plus gemcitabine	II	Locally advanced PD	Tolerability and activity for locally advanced PC were reported	[153]

**Table 2:** Marine-based drug delivery systems for pancreatic cancer treatment.

System	Drug	Cell Type/Animal Model	Main Outcomes	Ref.
Chitosan/glyceryl monooleate NPs	Gemcitabine	- BxPC-3 - MIA-PaCa-2	Drug-loaded NPs increased GEM accumulation and enhanced cytotoxic effects more than the naïve drug.	[161]
Herceptin-conjugated gemcitabine-loaded chitosan NPs (HER2-Gem-CS-NPs)	Gemcitabine	- MIA-PaCa-2 - PANC 1	The targeting capability of the developed system was validated. Increased cytotoxic effects were observed when compared with free drug and unconjugated NPs	[162]
Anti-EGFR Glycol-chitosan NPs loaded with gemcitabine ((Abc)-GC-Gem)	Gemcitabine	- SW1990	(Abc)-GC-Gem NPs targeted and inhibited cancer cell proliferation.	[163]
Folate-chitosan-gemcitabine core-shell NPs (FA-Chi-Gem)	Gemcitabine	- COLO357 - Orthotopic xenograft mice model	NPs inhibited pancreatic cancer cell proliferation and impaired tumour growth in vivo	[164]
Metformin encapsulated O-Carboxymethyl chitosan NPs (O-CMC-met)	Metformin	- MIA-PaCa-2 - Mice model	NPs downregulated gene expression (p21, vanin 1, and MMP9) in pancreatic cancer cells. There was a normal distribution of the NPs in vivo and no adverse effects over major organs	[165]
Chitosan-coated solid-lipid NPs	Aspirin Curcumin Sulforaphane	- BALB/c mice	No changes in blood counts, no abnormalities in different organs, and no toxicities	[166]
Chitosan NPs	Quercetin 5-fluorouracil	- MIA-PaCa-2	The encapsulation of both drugs increased cytotoxicity more than single-loaded NPs	[167]
Chitosan and PEG-coated curcumin-loaded Poly d,l-lactide-co-glycolide	Curcumin	- PANC-1 - MIA-PaCa-2	In vitro results demonstrated that NPs enhanced the cellular uptake, cytotoxicity, pro-apoptotic, anti-migratory, and anti-invasive properties as compared to free drugs	[168]
Fucoidan-coated manganese dioxide nanoparticles (Fuco-MnO_2_-NPs)	-	- AsPC-1 - BxPC-3 - BxPC3 xenograft mouse model	Fuco-MnO_2_-NPs and RT resulted in a greater tumour growth delay than RT alone	[169]
Fucoidan/lactoferrin NPs	-	- PANC-1	NPs increased the cytotoxicity and prevented the migration and invasion of pancreatic cancer cells	[175]
Photosensitizer-encapsulated amphiphilic sodium alginate derivative (Photosan-CSAD)	-	- PANC-1	NPs increased fluorescence activity and ROS generation, resulting in strong phototoxicity	[170]
Fibres-spun from alginate or chitosan loaded with gemcitabine	Gemcitabine	- MIA-PaCa-2 - PANC-1	Demonstrated a decrease in cells viability when compared with control fibres (no-gemcitabine)	[171]

## Data Availability

Not applicable.
